# Safeguarding transcriptional memory: a mitotic bookmarking role for chromatin remodelers

**DOI:** 10.1038/s41392-023-01610-5

**Published:** 2023-10-04

**Authors:** Godwin Sokpor, Huu Phuc Nguyen, Tran Tuoc

**Affiliations:** 1 Department of Human Genetics, Ruhr University of Bochum, Bochum, Germany; 2 Lincoln Medical School, University of Lincoln, Lincoln, UK

**Keywords:** Epigenetics, Epigenetic memory

**Fig. 1:**
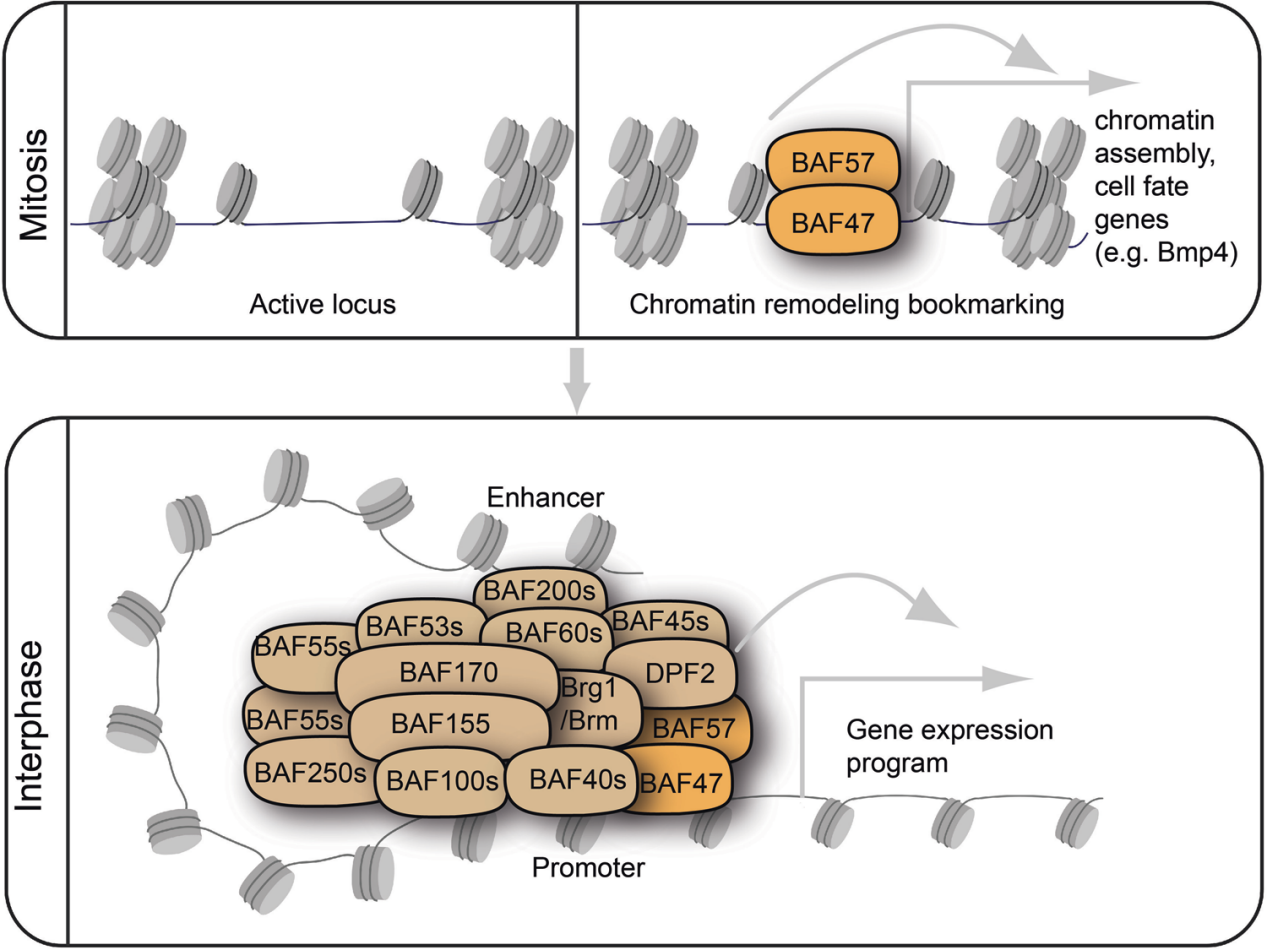
Schematic shows the regulation of chromatin bookmarks by the SWI/SNF (BAF) complex. During mitosis, limited chromatin sites remain active. Such active locus is a docking site for the specific SWI/SNF subunits SMARCB1 (BAF47) and SMARCE1 (BAF57) preferentially enriched at the promoter region of genes to establish a kind of gene bookmarking. Genes bookmarked at mitosis are vital for chromatin assembly and cell fate commitment (upper panel). The precise transmission of mitotic chromatin bookmarks constitutes an essential mechanism for transcriptional memory inheritance to preserve (re)activation of genes needed to be expressed after mitosis, e.g., at interphase under the control of wholly formed SWI/SNF (BAF) complexes (lower panel). The illustration was done using BioRender

A recent study published in *Nature* reveals an interaction between SWI/SNF subunits and mitotic chromatin, which is essential for establishing gene bookmarks and preserving cell identity.^1^ The findings deepen our understanding of how SWI/SNF complexes regulate transcriptional memory heritability for cell fate commitment (Fig. 1), thus validating the indispensability of SWI/SNF complexes in development and association of their perturbance with tumorigenesis and neurodevelopment disorders.^1,2^

The identity of cells and their propensity to acquire new physiological fate is critically checked at the transcriptional level to safeguard cell lineage. How the fidelity of regulatory mechanisms and their inheritability are ensured during the developmental progression of stem cells is unclear. The concept of mitotic bookmarking has evolved to delineate the genetic and molecular modalities for maintaining cell identity and fate during mitosis.^3^

The SWI/SNF or BAF complexes are chromatin remodelers assembled on a modular basis and in a context-specific manner to dictate gene expression patterns that influence cell state and function.^4,5^ As a result, SWI/SNF complexes or their subunits have been identified to be essential for many developmental processes, particularly neurodevelopment. Hence, certain neurological diseases and cancers have been linked to the dysfunction of SWI/SNF complexes.^2^ SMARCB1 and SMARCE1, also known as BAF47 and BAF57, respectively, are core subunits of the SWI/SNF complexes, namely the canonical BAF (cBAF) and polybromo-associated BAF (PBAF) complexes that were found bound to mitotic chromatin.^1,4^ Like many transcription factors, SWI/SNF subunits are expected to be degraded during mitosis.^3^ It is thus reasonable that SWI/SNF subunits that interact with chromatin in dividing cells may have implications for their participation in fate commitment or propagation of transcriptional blueprint. This idea may have motivated the curiosity of Zhu and colleagues to interrogate how the inheritance of somatic phenotypes is underscored by the gene expression modulatory activity of SWI/SNF subunits that linger at mitosis.^1^

Zhu et al.^1^ employed a biochemical fractionation strategy to investigate the presence, subcellular localization, and interaction of SWI/SNF complexes with chromatin in synchronized mouse embryonic stem cells spanning the cell cycle. It was identified that SMARCB1 and SMARCE1 were bound to mitotic chromatin, whereas other SWI/SNF subunits, including SMARCA4 (Brg1), were disengaged or residually bound. They subsequently used live cell imaging to substantiate the observation by in situ visualization of mitotic chromatin localization of SMARCB1 and SMARCE1 alongside SOX2, a classic mitotic chromatin-bound factor. The outcome of chromatin immunoprecipitation and sequencing, which was tweaked by Cut & Run sequencing approach, revealed the promoter region as the preferential genomic location of SMARCB1 and SMARCE1 in mitotic cells (Fig. 1). A high concentration of localized SMARCB1 and SMARCE1 at a subset of promoters are rationalized to be indicative of the involvement of target genes vital for the sustenance and transmission of transcriptomic traits during mitosis. However, it turned out SMARCE1 is not required for transcription maintenance because, despite its early expression in G1, it remains active later in the cell cycle. By probing the consequence of blocking SMARCE1 at mitosis, it became evident that mitotic SMARCE1 is essential for reactivating postmitotic genes. This provides formidable evidence for SMARCE1’s involvement in controlling cell fate through the reactivation of bookmarked genes in dividing cells, which function may be mutually exclusive of its traditional role in transcription regulation.^1^

A notable consequence of deactivating mitotic SMARCE1 is the downregulation of Bmp4, a suppressor of neural fate commitment. SMARCE1-deficient embryonic stem cells were hence observed to undergo precocious neurodifferentiation following neural induction.^1^ The phenotype was rescued by the BMP4 supplement.^1^ Thus, *Bmp4* bookmarking by SMARCE1 (Fig. 1) in embryonic or neural stem cells may be indispensable for establishing and subsequent activation of the transcriptional memory required for proper neural cell specialization or fate switch during neurodevelopment. To substantiate the importance of SMARCE1 functionality in neural development, we draw attention to the phenomenon of aberrant neural differentiation caused by SMARCE1 missense mutation leading to debilitating neurological disorders such as Coffin-Siris syndrome.^2^ The neurodevelopment implication can be widened if the argument covers the essence of mitotic docking of SMARCB1 and other SWI/SNF subunits.

Given that chromatin assembly genes were observed to be enriched for SMARCE1 binding, it can be inferred that SMARCE1 plays an early core function in the organization of whole SWI/SNF complexes. Indeed, it was identified that the whole SWI/SNF function is established after ATPase core recruitment in G1 is triggered by SMARCE1 and SMARCB1 mitotic docking.^1^ This corroborates the reasoning that during mitosis, rather than whole SWI/SNF complexes, a specific set of subunits bind to mitotic chromatin for bookmarking and priming of selected genomic regions for engagement of whole SWI/SNF complex function later in the cell cycle (Fig. 1).

The current work of Zhu et al.^1^ deepens our understanding of how the SWI/SNF complexes remodel the transcriptional landscape in mitosis by means of specific subunits binding to mitotic chromatin to bookmark genes therein, and such subunits drive the formation of whole SWI/SNF complexes later in the cell cycle. The identified chromatin bookmarking role for SMARCB1 and SMARCE1, interaction with SOX2, and gene expression regulation of Bmp4 by SMARCE1 describe a previously unknown important SWI/SNF subunit-mediated mitotic bookmarking mechanism for maintaining transcriptome integrity and ensuring the fidelity of cell identity inheritance (Fig. 1).

For future work, it would be interesting to probe the plausible contribution of SMARCC1 (BAF155) in mitotic bookmarking as it was observed bound to mitotic chromatin, although at a mild intensity.^1^ Including an investigation of other core subunits like BAF60 variants (SMARCD1/2/3) and BAF170 (SMARCC2) as the earliest assembled subunits of SWI/SNF complex^4,5^ may benefit expanding the range of SWI/SNF subunits involved in mitotic bookmarking and transmission of the transcriptional memory in cell cycle stages. Knowing the expression dynamics of SWI/SNF subunits driving gene bookmarking in the M-phase of lineage stem cells and cancer cells can lend therapeutic information.
